# Association of epicardial fat thickness with left ventricular diastolic function parameters in a community population

**DOI:** 10.1186/s12872-021-02071-w

**Published:** 2021-05-28

**Authors:** Wei Ma, Baowei Zhang, Ying Yang, Litong Qi, Jin Zhou, Min Li, Jia Jia, Yan Zhang, Huo Yong

**Affiliations:** 1 Department of Cardiovascular Disease, Peking University First Hospital, Beijing, China; 2 Key Laboratory of Molecular Cardiovascular Sciences (Peking University), Ministry of Education, Beijing, China; 3 Echocardiography Core Lab, Institute of Cardiovascular Disease at Peking, University First Hospital, Beijing, China; 4 Division of Cardiology, Peking University First Hospital, Dahongluochang Street, Xicheng District, Beijing, 100034 China

**Keywords:** Epicardial fat, Thickness, Left ventricle, Diastolic function

## Abstract

**Background:**

We examined the relationship between epicardial fat thickness (EFT) measured by echocardiography and left ventricular diastolic function parameters in a Beijing community population.

**Methods:**

We included 1004 participants in this study. Echocardiographic parameters including E and A peak velocity, the early diastolic velocities (e′) of the septal and lateral mitral annulus using tissue doppler imaging, E/e′, and EFT were measured. EFT1 was measured perpendicularly on the right ventricular free wall at end diastole in the extension line of the aortic root. EFT2 was the maximum thickness measured perpendicularly on the right ventricular free wall at end diastole. Multivariable linear regression was used to analyze the relationship between EFT and the mean e′ and E/e′.

**Results:**

The mean age of the participants was 63.91 ± 9.02 years, and 51.4% were men. EFT1 and EFT2 were negatively correlated with lateral e′, septal e′, and mean e′ (*p* < 0.05), and the correlation coefficient for EFT1 and EFT2 and mean e′ was − 0.138 and − 0.180, respectively. EFT1 and EFT2 were positively correlated with lateral E/e′, septal E/e′, and mean E/e′ (*p* < 0.05), and the correlation coefficient for EFT1 and EFT2 and mean e′ was 0.100 and 0.090, respectively. Multivariable egression analysis showed that EFT2 was independently and negatively associated with e′ mean (β = − 0.078 [95% confidence interval = − 0.143, − 0.012, *p* = 0.020]). There were no interactions between EFT2 and any covariates, including age or heart groups, sex, BMI, or presence of hypertension, diabetes, or coronary heart disease, in relation to left ventricular diastolic dysfunction.

**Conclusions:**

EFT2 was negatively and independently associated with e′ mean, which suggests that more attention to this type of adipose fat is required for cardiovascular disease therapy.

## Introduction

The incidence of obesity has progressively risen with the increasing prevalence of unhealthy lifestyles in recent years. An increase in visceral fat is the main manifestation of the abnormal fat distribution in obesity, which is closely related to cardiovascular disease and metabolic syndrome [1]. Therefore, abnormal fat distribution should be taken into account when considering cardiovascular risk assessment.

Two types of fat are present around the heart: epicardial and pericardial fat [2]. Epicardial fat is located between the myocardium and visceral pericardium. Pericardial fat is located on the external surface of the parietal pericardium [2]. Epicardial fat is an important endocrine and paracrine organ that produces a variety of active substances [2]. Epicardial fat is closely associated with obesity, metabolic syndrome, and heart disease, especially coronary artery disease (CAD) [3], and can predict the development of cardiovascular disease and mortality in patients with type 2 diabetes [4].

Left ventricular (LV) diastolic dysfunction is one of the main factors associated with all-cause mortality [5]. Obesity is an important risk factor for heart failure with preserved LV ejection fraction (LVEF) [6], and intramyocardial fat is correlated with LV diastolic dysfunction in these patients [7]. Further, an increased epicardial fat thickness (EFT) is associated with LV diastolic function impairment in patients with obesity and coronary heart disease (CHD) [8–10]. However, community population research is still lacking. Such research may lead physicians to pay closer attention to the epicardial fat status in community populations, not only in high-risk populations. Thus, we examined the relationship between EFT as measured by echocardiography and LV diastolic function parameters in a Beijing community population.

## Methods

### Population

All residents who were 40 years of age or older and lived in Shi Jing Shan district of Beijing were invited. Residents were contacted by recruitment advertisements or by telephone, and those volunteering to participate were included. Of the 5593 individuals who were ≥ 40 years of age, 1058 (18.9%) volunteered to participate in this study. The investigation started in July 2004 and ended in June 2005. Participants who had echocardiography were recruited in this study, while participants who had valve disease, evident arrhythmia (including atrial fibrillation), regional wall movement abnormal, or LVEF < 50% were excluded. Finally, 1004 participants were included in this study. The study was approved by the institutional review board of Peking University First Hospital, and informed consent was obtained from all participants.

### Definition of cardiovascular risk factors and disease

Waist circumference (WC) was measured in the standing position using the middle circumference between the lower rib margin and the iliac crest. The body mass index (BMI) was also calculated. After a rest period of 15 min, blood pressure (BP) was measured three times, with 5-min intervals between each measurement, at the right upper arm in a sitting position with a mercury sphygmomanometer. Mean systolic BP and diastolic BP were calculated on the basis of the three measurements. The total cholesterol and total triglyceride concentrations were analyzed by a fasting blood sample using standard techniques at the Beijing Hypertension League Institute. Participants with cigarette smoking history and current smokers were identified as smokers. Hypertension was defined as office systolic BP ≥ 140 mmHg and/or diastolic BP ≥ 90 mmHg, or history or the usage of antihypertensive drugs. Diabetes was diagnosed according to each participant’s interview and use of hypoglycemic drugs. Participants with a fasting glucose ≥ 7.0 mmol/L and a 2-h glucose ≥ 11.1 mmol/L on the oral glucose tolerance test were also defined as diabetic. Stroke, including intracerebral hemorrhage, cerebral infarction, and transient ischemic attack, was determined on the basis of a history of data collected from hospitalizations and outpatient records, which were confirmed by computed tomography (CT) or magnetic resonance imaging (MRI) scan [11]. A history of old myocardial infarction, percutaneous coronary intervention, and coronary artery bypass grafting were all included in CHD.

### Echocardiography

Echocardiography was performed using a 3-MHz transducer in an ultrasound system (Vivid-7; General Electric). According to the guidelines [12], standard images were collected and stored. One experienced echocardiography doctor blinded to the clinical picture of the participants measured the echo parameters in Peking University First Hospital central lab.

LV internal dimensions, LV wall thickness, and LVEF (measured by the Teichholz method) were based on the guidelines established by the American Society of Echocardiography [12]. LV mass (LVM) was calculated as LVM = 0.8 × 1.04 × ([PWTd + VSTd + LVIDd]^3^ − [LVIDd]^3^) + 0.6 g, where PWTd and SWTd are the posterior and septal wall thickness at end diastole, respectively, and LVIDd is the LV dimension at end diastole [12]. LV mass index (LVMI) was calculated. The biplane method was used to measure the maximum left atrial volume (LAV), and LAV index (LAVI) was calculated. Transmitral inflow velocities were measured using pulsed doppler at the mitral valve leaflet tips in the apical four-chamber view. The E wave velocity, A wave velocity, E/A ratio, and E wave deceleration time (DT) were measured. Tissue Doppler imaging was used to measure LV myocardial velocities in the apical four-chamber view, and the early diastolic velocities (e′) of the septal and lateral mitral annulus were measured. The mean e′ was calculated as the mean of the septal e′ (e′ sep) and lateral e′ (e′ lat). The E/e′ was calculated, and the mean E/e′ was calculated as the mean of the septal E/e′ (E/e′ sep) and lateral E/e′ (E/e′ lat) regions. The mean Doppler values were obtained over three different cardiac cycles.

EFT was identified as the echo-free space between the myocardium and the visceral layer of the pericardium from the parasternal long axis view. EFT1 was measured in the extension line of the aortic root and perpendicularly on the right ventricular free wall. EFT2 was the maximum thickness measured perpendicularly on the right ventricular free wall. Both EFTs were measured at end-diastole. The intra class correlation coefficient was used to evaluate intra-researcher and inter-researcher consistency. Interobserver values for EFT1 and EFT2 were 0.897 (95% confidence interval [CI]: 0.794–0.949, *p* < 0.05) and 0.914 (95% CI: 0.827–0.958, *p* < 0.05), respectively, while intraobserver values for EFT1 and EFT2 were 0.532 (95% CI: 0.218–0.746, *p* < 0.05) and 0.548 (95% CI: 0.240–0.756, *p* < 0.05), respectively.

### Statistical analysis

Continuous data are presented as mean ± standard deviation, while frequency data are presented as percentages. Spearman correlation was used to analyze the correlation of EFT with echocardiographic parameters. Multivariable linear regression was used to analyze the relationship between EFT and e′ mean and E/e′ mean, adjusting for age, heart rate, WC, hypertension, diabetes, and CHD. Subgroup analyses and interaction tests were used to examine the EFT2 and e′ mean according to age (< 65 years or ≥ 65 years), sex (male or female), BMI (< 28 kg/m^2^ or ≥ 28 kg/m^2^), heart rate (< 80 beats per min or ≥ 80 beats per min), hypertension (yes or no), diabetes mellitus (yes or no), and CHD (yes or no). A *p* value < 0.05 (two-sided) was considered statistically significant for all tests. All analyses were performed with statistical software (Empower(R), www.empowerstats.com; X&Y solutions, Inc., Boston, MA, USA; R [http://www.R-project.org] v3.4.3; SPSS v13.0).

## Results

The general characteristics of the participants are shown (Table 1). The mean age was 63.91 ± 9.02 years of age, and 51.4% of participants were male. The prevalences of hypertension, diabetes, CHD, and stroke were 80.0%, 29.4%, 12%, and 16.1%, respectively. The echocardiography parameters of the participants are shown in Table 2. The e′ lat, e′ sep, and e′ mean were all reduced compared with normal values. There were no changes in indicators of LV filling pressure (E/e′ sep, E/′ lat, and E/e′ mean).

**Table 1:** General characteristics of the study participants

Variables	Values
Sex (male, n, %)	516 (51.4)
age (years)	63.91 ± 9.02
BMI (kg/m^2^)	25.88 ± 3.40
WC (cm)	88.72 ± 9.21
SBP (mmHg)	133.98 ± 17.95
DBP (mmHg)	80.07 ± 10.97
HR (beats/min)	71.09 ± 10.04
Smoking (n, %)	352 (35.0)
Hypertension (n, %)	803 (80.0)
Diabetes (n, %)	295 (29.4)
CHD (n, %)	120 (12.0)
Stroke (n, %)	162 (16.1)
TC (mmol/l)	5.28 ± 1.02
TG (mmol/l)	2.30 ± 1.92
Fast Glucose (mmol/l)	6.36 ± 2.24

*BMI* body mass index, *WC* waist circumference, *SBP* systolic blood pressure, *DBP* diastolic blood pressure, *HR* heart rate, *CHD* coronary heart disease, *TC* total cholesterol, *TG* total triglyceride

**Table 2:** Echocardiography parameters of the participants

Variables	Values
IVS (cm)	0.94 ± 0.13
LVPW (cm)	0.92 ± 0.07
LVEDD (cm)	4.30 ± 0.50
LVESD (cm)	2.65 ± 0.90
LVMI (g/m^2^)	76.91 ± 16.20
LAVI (ml/m^2^)	34.14 ± 9.83
E peak velocity (cm/s)	78.41 ± 17.91
A peak velocity (cm/s)	89.12 ± 18.39
DT (ms)	245.09 ± 48.22
E/A	0.91 ± 0.27
e′ lat (cm/s)	9.37 ± 2.73
e′ sep (cm/s)	6.54 ± 2.04
e′ mean(cm/s)	7.96 ± 9.83
E/e′ lat	8.99 ± 3.18
E/e′ sep	12.83 ± 4.02
E/e′ mean	10.91 ± 3.26
LVEF (%)	69.11 ± 8.25
EFT1 (cm)	0.39 ± 1.26
EFT2 (cm)	0.68 ± 1.77

*IVS* interventricular septum thickness, *LVPW* left ventricular posterior wall thickness, *LVEDD* left ventricular end diastolic diameter, *LVESD* left ventricular end systolic diameter, *LVMI* left ventricular mass index, *LAVI* left atrial volume index, *DT* E wave deceleration time, *e′ sep* septal e′, *e′ lat* lateral e′, *E/e′ lat* lateral E/e′, *E/e′ sep* septal E/e′, *LVEF* left ventricular ejection fraction, *EFT1* epicardial fat thickness 1 (measured perpendicularly on the right ventricular free wall at end-diastole in the extension line of the aortic root), *EFT2* epicardial fat thickness 2 (the maximum thickness measured perpendicularly on the right ventricular free wall at end-diastole)

EFT1 and EFT2 were positively correlated with interventricular septum thickness and LV posterior wall thickness (*p* < 0.05), but not with LVMI and LAVI (*p* > 0.05). EFT1 and EFT2 were negatively correlated with E and E/A (*p* < 0.05), and positively correlated with A and DT (*p* > 0.05). EFT1 and EFT2 were negatively correlated with e′ lat, e′ sep, and e′ mean (*p* < 0.05), and positively correlated with E/e′ lat, E/e′ sep, and E/e′ mean (*p* < 0.05) (Table 3).

**Table 3:** Correlation analysis of epicardial fat thickness (EFT) with echocardiography parameters

	EFT1	EFT2
IVS	0.108^a^	0.124^a^
LVPW	0.115^a^	0.136^a^
LVEDD	− 0.039	− 0.060
LVESD	− 0.012	− 0.019
LVMI	0.030	0.016
LAVI	− 0.029	− 0.049
E peak velocity	− 0.070^a^	− 0.113^a^
A peak velocity	0.121^a^	0.107^a^
DT	0.073^a^	0.089^a^
E/A	− 0.154^a^	− 0.206^a^
e′ lat	− 0.136^a^	− 0.162^a^
e′ sep	− 0.129^a^	− 0.146^a^
e′ mean	− 0.138^a^	− 0.180^a^
E/e′ lat	0.093^a^	0.090^a^
E/e′ sep	0.097^a^	0.081^a^
E/e′ mean	0.100^a^	0.090^a^
LVEF	0.002	− 0.009

^a^*p* < 0.05

*IVS* interventricular septum thickness, *LVPW* left ventricular posterior wall thickness, *LVEDD* left ventricular end diastolic diameter, *LVESD* left ventricular end systolic diameter, *LVMI* left ventricular mass index, *LAVI* left atrial volume index, *DT* E wave deceleration time, *e′ sep* septal e, *e′ lat* lateral e′, *E/e′ lat* lateral E/e′, *E/e′ sep* septal E/e′, *LVEF*, left ventricular ejection fraction

**Fig. 1:**
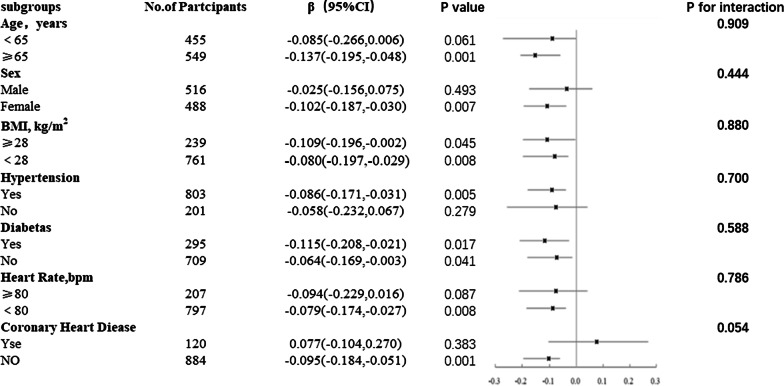
Subgroup analysis of the relationship between EFT2 and e′ mean adjusted for age, sex, heart rate, WC, hypertension, diabetes, and coronary heart disease

Univariatable analysis showed that EFT1 and EFT2 were negatively associated with e′ mean, while multivariable regression analysis showed that EFT2 was negatively associated with e′ mean after adjusting for age, sex, heart rate, WC, hypertension, diabetes, and CHD. EFT1 and EFT2 were not independently related with E/e′ mean on univariatable analysis or multivariable regression analysis (Table 4). Results of subgroup analysis of the relationship between e′ mean and EFT2 are shown in Fig. 1. There were no interactions between EFT2 and any covariates, including age and heart groups, sex, BMI, or presence of hypertension, diabetes, or CHD. These findings were consistent with EFT1 (data not shown).

**Table 4:** Univariable and multivariable regression analysis of epicardial fat thickness (EFT) and e′ mean and E/e′ mean

	e′ mean				E/e′ mean			
	Crude β (95%CI)	*p*	Adjusted β (95%CI)	*p*	Crude β (95%CI)	*p*	Adjusted β (95%CI)	*p*
EFT1	− 0.235 (− 0.341, − 0.130)	< 0.001	− 0.089 (− 0.177, − 0.000)	0.050	0.212 (0.052, 0.372)	0.010	0.035 (− 0.116, 0.186)	0.652
EFT2	− 0.222 (− 0.297, − 0.146)	< 0.001	− 0.078 (− 0.143, − 0.012)	0.020	0.181 (0.066, 0.296)	0.002	0.012 (− 0.099, 0.123)	0.829

Adjusted for age, sex, heart rate, WC, hypertension, diabetes, and CHD

## Discussion

Many studies have focused on the association of adipose tissue and LV diastolic dysfunction, including global adiposity [13], central adiposity [14], and visceral adiposity [15]. Epicardial fat plays an important role in lipid and energy metabolism, and this fat can also have harmful effects because it can secrete many proatherogenic and proinflammatory cytokines [16]. In the present study, EFT measured by echocardiography was independently associated with e′ mean, but not with E/e′ mean, in a high-risk community population with an LVEF of ≥ 50%. Subgroup analysis showed there were no interactions between EFT and any covariates, including age and heart groups, sex, BMI, or presence of hypertension, diabetes, or CHD.

Epicardial fat can be assessed by multiple imaging methods, including CT, MRI, and echocardiography. The thickness, area, and volume of epicardial fat can be measured by CT with manual or semi-automated methods. However, the high cost and radiation exposure associated with CT are disadvantageous, especially in large population studies. MRI is considered the gold standard for evaluating heart fat, although its use is also limited because of high cost and high requirement. Conversely, echocardiography is the most convenient method to evaluate epicardial fat, and is particularly suitable for epidemiological studies. The thickness and area of the epicardial fat can be measured with echocardiography [3], although the image quality has a marked influence on accuracy. Further, there can be differences between different readers. We used echocardiography to evaluated EFT in the present study. Echocardiography is widely used to evaluate epicardial fat [3]. Using ultrasound measurements, a Korean study reported a correlation between EFT and CT measurements of epicardial fat volume [17]. Echocardiographic epicardial fat measurements were also shown to have a strong correlation with MRI measurements [18]. It is important to note that paracardial fat consists of epicardial fat and pericardial fat, which should be distinguished during echocardiography examination. Echocardiographic EFT can be measured from the parasternal long and short axis views. However, EFT measured from the long axis view (but not the short axis view) was reported to be the independent predictor of e′ septal and e′ lateral [19].

In the present study, EFT was associated with most echocardiographic parameters. For example, EFT was positively correlated with ventricular septum and LV posterior wall thickness, but not with LVMI. One study showed that an increasing epicardial fat thickness was significantly related to an increase in LVM because of high free fatty acids levels, insulin resistance and adrenergic activity, and that increased visceral fat directly affected LV output to perfuse the increased body mass [20]. In that study, the participants were younger (mean age, 46.9 years) and had a higher BMI (30–30.5 kg/m^2^) than in our cohort. A further study reported that EFT was correlated with atria enlargement in morbidly obese subjects [8]. Conversely, we found no associated of EFT with LAVI, although our participants were older, with a higher prevalence of hypertension. Thus, LVMI and LAVI may be more significantly correlated with hypertension and age.

Several echocardiographic parameters used to evaluate LV diastolic function. Mitral inflow patterns, including E and A wave velocity, E/A, and E wave DT, are affected by many factors. A significant association of e′ with LV relaxation was reported in human subjects [21]. The E/e′ ratio can also be used to evaluate LV filling pressures [22]. Further, e′ is a powerful predictor of cardiac mortality in patients, independent of normal or abnormal LV systolic function [23, 24], while mitral E/e′ is a strong predictor of cardiac death or rehospitalization for CHF as well[25]. In our correlation analysis, EFT was negatively associated with E, E/A, and e′ and positively associated with A, DT, and E/e′. In multivariate regression analysis, EFT was independently associated with e′ mean but not with E/e′. Interestingly, in Japanese patients with known or suspected CAD, EFT was negatively associated with e′ mean and positively correlated with E/e′ mean [10]. Konishi et al. also reported that epicardial fat volumes measured by CT were significantly and independently associated with E/e′ > 10 in suspected CAD patients [15]. Further EFT was significantly associated with LV diastolic dysfunction in subjects with normal coronary artery [26]. Finally, Dabbah et al. reported that E/e′ was not associated with EFT [19], similar to that in the present study.

In our subgroup analysis, there was no effect of CHD on the relationship between EFT2 and e′ mean. Cavalcante and Konishi reported an independent association of epicardial fat and E/e′, although some patients may have advanced diastolic dysfunction since ischemia because that study included patients suspected of CAD [15]. Hypertension is a risk factor for the occurrence of LV diastolic dysfunction. In patients with newly diagnosed and untreated hypertension, increased EFT was significantly and independently related to the degree of LV diastolic function [9]. In our subgroup analysis, we found no effect of hypertension on the relationship between EFT2 and e′ mean. It was also reported that EFT was more common in women than men > 60 years old, and that EFT was significantly related to LV function in women, but not men [27]. Conversely, we found no interactions of different ages or sex on the association of EFT with e′ mean. All of these contrasting findings may be related to the different imaging methods for evaluating epicardial fat, or to different populations of patients.

There are several limitations to our study. First, epicardial fat was measured by echocardiography rather than MRI or CT. However, EFT measured by echocardiography was shown to correlate with volumetric measurements. Second, because of the cross-sectional nature of our study, a causal relationship between EFT and e′ cannot be determined. Prospective studies examining whether increased EFT is predictive of LV diastolic dysfunction are required. Finally, most patients were > 40 years of age, and thus our findings may not reflect the characteristics of epicardial fat in a younger population.

In summary, EFT measured by echocardiography was independently correlated with the e′ mean, but not with the E/e′ mean, in a high-risk community population with an LVEF ≥ 50%. Subgroup analysis showed there were no interactions between EFT and any covariates, including age and heart groups, sex, BMI, or presence of hypertension, diabetes, or CHD.

## Data Availability

The datasets generated and/or analyzed during the current study are not publicly available due to the government policy but are available from the corresponding author on reasonable request.
